# Ongoing Spontaneous Activity Controls Access to Consciousness: A Neuronal Model for Inattentional Blindness

**DOI:** 10.1371/journal.pbio.0030141

**Published:** 2005-04-12

**Authors:** Stanislas Dehaene, Jean-Pierre Changeux

**Affiliations:** **1**INSERM-CEA Unit 562, Cognitive NeuroimagingService Hospitalier Frédéric Joliot, OrsayFrance; **2**CNRS URA2182 Récepteurs and Cognition, Institut PasteurParisFrance; Brandeis UniversityUnited States of America

## Abstract

Even in the absence of sensory inputs, cortical and thalamic neurons can show structured patterns of ongoing spontaneous activity, whose origins and functional significance are not well understood. We use computer simulations to explore the conditions under which spontaneous activity emerges from a simplified model of multiple interconnected thalamocortical columns linked by long-range, top-down excitatory axons, and to examine its interactions with stimulus-induced activation. Simulations help characterize two main states of activity. First, spontaneous gamma-band oscillations emerge at a precise threshold controlled by ascending neuromodulator systems. Second, within a spontaneously active network, we observe the sudden “ignition” of one out of many possible coherent states of high-level activity amidst cortical neurons with long-distance projections. During such an ignited state, spontaneous activity can block external sensory processing. We relate those properties to experimental observations on the neural bases of endogenous states of consciousness, and particularly the blocking of access to consciousness that occurs in the psychophysical phenomenon of “inattentional blindness,” in which normal subjects intensely engaged in mental activity fail to notice salient but irrelevant sensory stimuli. Although highly simplified, the generic properties of a minimal network may help clarify some of the basic cerebral phenomena underlying the autonomy of consciousness.

## Abstract 2

Computer simulations of the circuits of activity in the cerebral cortex and underlying thalamus suggest that precisely controlled oscillatory states can control the central neural processing of sensory information

## Introduction

Ongoing spontaneous activity is present throughout the nervous system [1], but its function remains enigmatic. In the embryo, spontaneous movements [2] and waves of endogenous retinal activity [3,4] are thought to play an important role in the epigenesis of neural networks through selective synapse stabilization [5,6]. Ongoing spontaneous activity is also present in the adult brain, where it is responsible for the highly variable patterns of the electroencephalogram (EEG). Thalamocortical networks generate a variety of oscillations whose rhythms change across the sleep-wake cycle [7,8,9]. Optical imaging methods in anesthetized animals also reveal fast spontaneous states of neuronal activity that, far from being random, exhibit patterns that resemble those evoked by external stimuli [10,11]. In parallel, functional neuroimaging studies in humans have shown a globally elevated brain metabolism at rest, with localized patterns suggesting that particular cortical regions are maintained in a high, although variable, state of activity [12,13,14,15,16]. At present, the functional roles of this spontaneous activity in the adult brain at rest remains to be elucidated.

In previous neuronal modeling studies and computer simulations, we illustrated the possible contribution of spontaneous activity to tasks that involve a random search, such as the learning of a temporal sequence [17], the search for and selection of the correct rule in the delayed response and Wisconsin card-sorting tests [18], or the discovery of a multistep solution in the Tower of London test [19]. More recently, generalizing from this early work, we proposed a broader framework of a formal architecture of thalamocortical areas, in which top-down activity generated in hierarchically higher cortical areas plays a key role in what we referred to as “access to consciousness” in an effortful task [20,21,22,23]. Like several previous proposals, our model of a conscious neuronal workspace distinguishes lower automatized systems from increasingly higher and more autonomous supervisory systems [24]. It also builds upon Baars' cognitive theory of consciousness, which distinguishes a vast array of unconscious specialized processors running in parallel, and a single limited-capacity serial “workspace” that allows them to exchange information [25].

The proposed neuronal architecture separates, in a first minimal description, two computational spaces, each characterized by a distinct pattern of connectivity. Subcortical networks and most of the cortex can be viewed as a collection of specialized and automatized processors, each attuned to the processing of a particular type of information via a limited number of local or medium-range connections that bring to each processor the “encapsulated” inputs necessary to its function. On top of this automatic level, we postulate a distinct set of cortical “workspace” neurons characterized by their ability to send and receive projections to many distant areas through long-range excitatory axons, thus allowing many different processors to exchange information.

Our previous simulations demonstrated how this architecture could account for a psychological phenomenon, the “attentional blink.” Because of its long-distance, brain-scale connectivity, the global workspace establishes a central processing bottleneck such that, in the presence of two competing stimuli, processing of the first temporarily blocks high-level processing of the second [22]. While this work simulated only sensory processing, a key hypothesis of the workspace model is that the neurons of the higher level, the workspace neurons, are the seat of a permanent spontaneous activity that creates a succession of active internal states [20,21,23]. The aim of the present paper is to explore in a more extensive and systematic manner the role of this ongoing spontaneous activity in a similar neural network comprising several nested levels of neuronal architecture. We propose a specific network architecture and perform explicit computer simulations that offer plausible explanations for the origins and function of structured spontaneous activity in adult thalamocortical circuits, and in particular its critical role in allowing or blocking access by sensory stimuli.

The observed dynamic properties of the network lead us to distinguish two main transitions in activation. First, a neuromodulatory substance is assumed to control the level of network activation; as its input increases continuously, the network exhibits a sudden surge in spontaneous activation and switches to a state of thalamocortical resonance characterized by temporary bouts of synchronized gamma-band oscillations of increasing amplitude. This state of activity leads to a facilitation of sensory processing, and is proposed to correspond to the state of vigilance or being awake.

When the simulated areas are reciprocally connected by long-distance excitatory connections, a second state transition can occur. A subset of areas may suddenly show a strong temporary increase in synchronized firing and form a coherent state of activity (“ignition”). The transition to this state of high correlated activity is fast and characterized by an amplification of local neural activation and the subsequent ignition of multiple distant areas. This state of activity competes with, rather than facilitates, sensory processing, and thus leads to an extinction of sensory processing. We propose that this blocking may account for the “inattentional blindness” phenomenon, in which normal subjects intensely engaged in mental activity fail to notice salient but task-irrelevant sensory stimuli [26,27,28].

## Results

We used computer simulations to characterize spontaneous and evoked activity in a complex nested architecture comprising multiple neurons, columns, and areas (Figure 1). To facilitate comprehension, we organize the results section as a progression from local to more global states of activity. We start by describing the spontaneous and evoked activity in the building blocks of the model, namely the single neuron and an isolated thalamocortical column. We then consider the extent to which those properties are affected when multiple thalamocortical columns are interconnected by long-distance, bottom-up and top-down connections.

**Figure 1:**
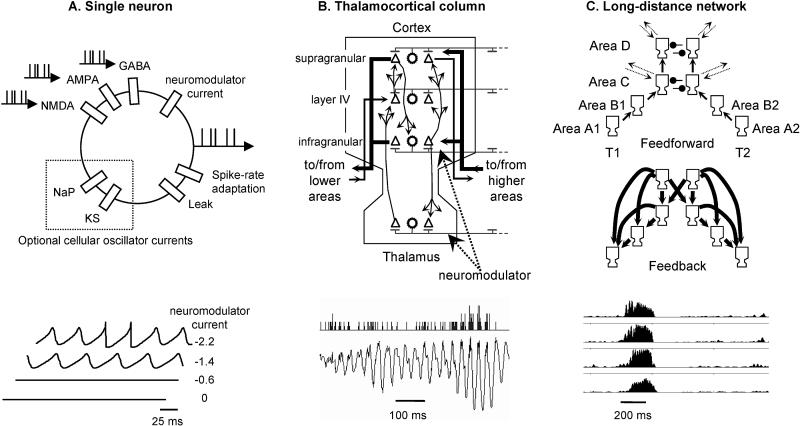
Simulation Components and Resulting Spontaneous Activity Shown are the constituents of the simulation (upper diagrams) and typical patterns of spontaneous activity that they can produce (lower tracings). We simulated a nested architecture in which spiking neurons (A) are incorporated within thalamocortical columns (B), which are themselves interconnected hierarchically by local and long-distance cortical connections (C) (see Materials and Methods for details). While single neurons may generate sustained oscillations of membrane potentials (A), only the column and network levels generate complex waxing-and-waning EEG-like oscillations (B) and metastable global states of sustained firing (C).

### Spontaneous Oscillatory Behavior in a Single Neuron and a Thalamocortical Column

We first simulated a single neuron using the “cellular oscillator” model derived from Wang [29]. The results appear in Figure 2A. In the absence of any depolarizing current, the resting membrane potential is stable at *V* ≈ −63 mV. Injection of an increasing depolarizing current *I*_neuromodul_ leads to the sudden emergence, at a rather precise value (*I*_neuromodul_≈ −1.1 μA/cm^2^), of oscillations in membrane potential in the gamma range. Two features characterize this transition as a supercritical Hopf bifurcation according to dynamical systems theory [30]. First, a discontinuous transition is observed in the frequency domain, with the oscillation emerging suddenly at a characteristic frequency of 30–35 Hz, and changing only slowly with increasing current (up to 40–45 Hz). Second, a continuous transition is observed in the amplitude domain, with oscillation amplitude increasing continuously from zero as the square root of the amount of deviation from the threshold current (and therefore power increases linearly, as shown in Figure 2A).

**Figure 2:**
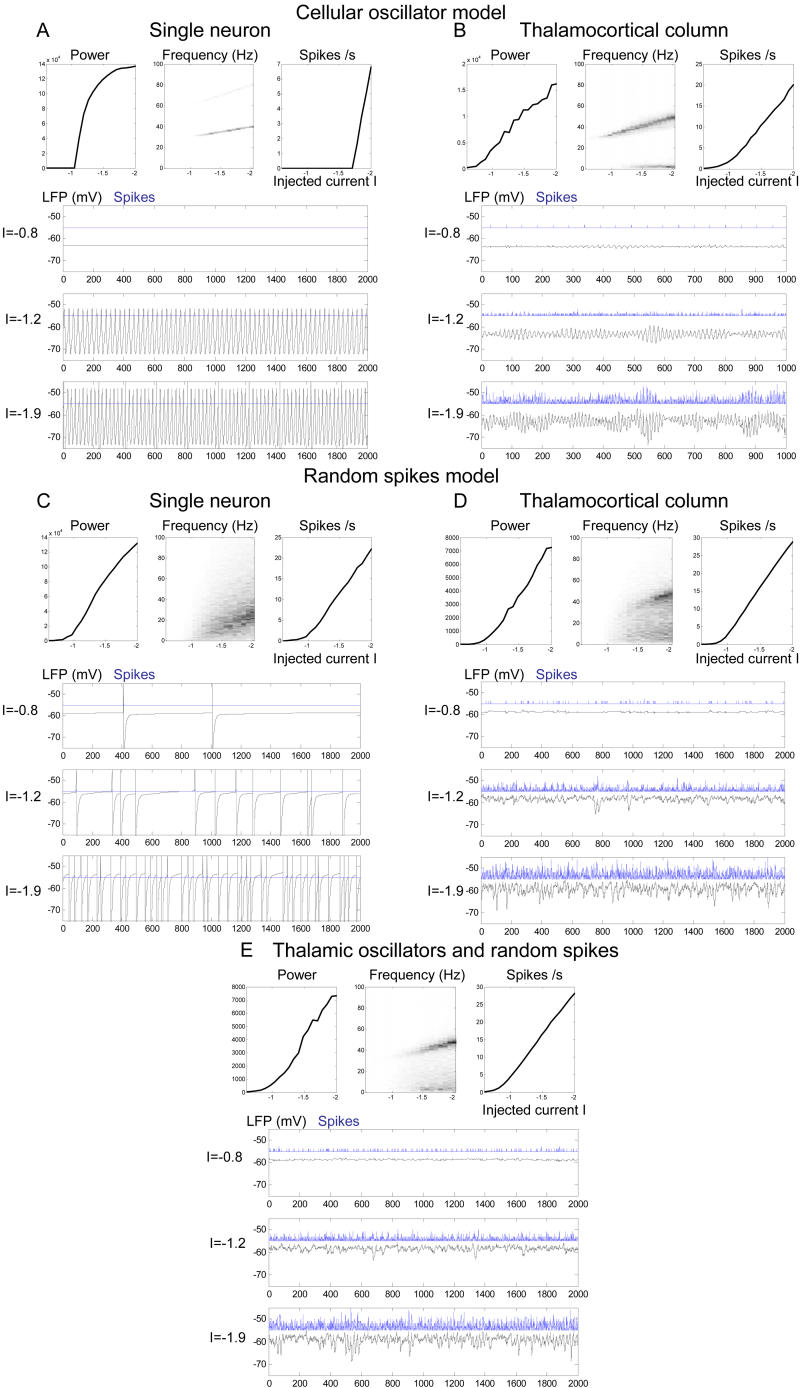
Characterizing the Transition Toward Spontaneous Activity In each panel, we show the evolution of the power of the local field potential (top left of each), frequency (top center), and mean firing rate of pyramidal neurons (top right) as a function of the injected neuromodulatory current *I*_neuromodul_. The elongated graphs give examples of the temporal evolution of the local field potential (bottom trace) and spikes (top trace) as a function of time in milliseconds for three different levels of *I*_neuromodul_ *.* The simulations were performed with a single neuron (A and C) or with a whole thalamocortical column (B, D, and E), and with different sources of spontaneous activity: intrinsic cellular oscillations (A and B), random spikes (C and D), or both random spikes and intrinsic thalamic oscillators (E). Regardless of the exact mechanism, the architecture of thalamocortical columns created a bifurcation characterized by the sudden emergence, at a relatively constant threshold current, of high-frequency oscillations in the gamma range (> 20 Hz), with continuously increasing power as a function of *I*_neuromodul_.

Around *I*_neuromodul_ ≈ −1.7 μA/cm^2^, a second threshold is observed: When oscillation amplitude reaches the voltage threshold for spiking, a spike is generated. Firing rate increases essentially linearly above this threshold.

Overall, those properties of the model are similar to the gamma-band (“40 Hz”) subthreshold membrane oscillations observed in intracellular recordings of thalamic and cortical neurons [7,31,32,33]. In both our simulations and these experiments, oscillations emerge at a precise depolarization threshold, with a sudden well-defined frequency and a continuously increasing amplitude.

Similar properties continued to be observed when 120 such oscillatory neurons, with randomized membrane parameters, were interconnected in a model thalamocortical column (as described in Materials and Methods). Figure 2B shows the temporal evolution of the average local field potential (LFP) emitted by the cortical excitatory neurons in response to variable levels of injected current *I*_neuromodul_. There is still a threshold, now lowered to *I*_neuromodul_ ≈ −0.8 μA/cm^2^, at which gamma-band oscillations emerge with a fixed frequency (initially 30–35 Hz) and with continuously increasing amplitude. The lowering of the threshold is due to random variability between neurons in the conductance of the Na^+^ and K^+^ channels responsible for generating membrane oscillations. Some neurons begin to oscillate at a lower value of the injected current, thus smoothing out the sharp transition observed within each single neuron. For the same reason, the spiking threshold is also lowered in this single-column simulation, with the number of emitted spikes increasing smoothly starting around *I*_neuromodul_ ≈ −0.9 μA/cm^2^.

In the absence of spikes, the membrane oscillations of different neurons are independent of each other. Spikes, however, introduce coupling and result in transient periods of synchrony, which appear as occasional increases changes in firing rate accompanied by high-amplitude LFPs and a strong synchronization of the thalamocortical column. Continuous plotting of the LFP reveals a spontaneous, semirandom waxing and waning of bouts of coherent gamma-band oscillations with typical durations of 100–150 ms (Figures 1B and 2B). The temporal evolution of the LFP appears largely chaotic and unpredictable, although the underlying simulation is strictly deterministic.

Altogether, these properties are comparable to the synchronized, depolarization-dependent, high-frequency thalamocortical oscillations that have been observed, for instance, in the cat thalamus and cortex [34,35]. For simplicity, we did not include mechanisms for sleep-related, low-frequency oscillations, which are not the focus of the present model but have been simulated by others [9,36].

### Thalamocortical Resonance without Intrinsic Oscillators

To evaluate the role of intrinsic cellular oscillators in generating the above thalamocortical oscillations, we reiterated the simulations using the “random spikes” model, with simplified neurons devoid of the Na^+^ and K^+^ channels, but with a random (Gaussian) moment-to-moment variability in spike initiation threshold. We verified that a single such passive, single-compartment, integrate-and-fire neuron, faced with a constant input current, is incapable of generating membrane oscillations below the spiking threshold (p. 163, [37]). In a single neuron, oscillations appear only once the injected neuromodulatory current is sufficient to depolarize the neuron beyond the spiking threshold. Even then, they do not exhibit a fixed central frequency, but cover a broad spectrum that progressively increases and broadens starting at 0 Hz, and stays mostly below 30 Hz with the present parameters (Figure 2C).

Despite those major differences at the single-unit level, when 120 such neurons were connected into a thalamocortical column architecture, we observed structured spontaneous activity and phase transitions analogous to those of the cellular oscillator model (Figure 2D; compare with Figure 2B). Once a sufficient number of neurons were depolarized above the spiking threshold, the local field potential began to wax and wane within a range of gamma-band frequencies. Although this band was initially rather broad, it quickly narrowed to a predominant band at 40–45 Hz for higher values of the injected current. As shown in Figure 2D, the critical properties of continuously increasing oscillation amplitude with a well-characterized frequency range remained, due no longer to intrinsic membrane properties, but to the temporal filtering properties of the several connection loops present in the thalamocortical column. Those loops have the effect of filtering random spiking activity, thus biasing neurons toward generating spikes at recurrent, rather randomly organized times. For instance, a major excitatory loop circles from the thalamus to the layer IV, supragranular, and infragranular cortical neurons, and finally back to the thalamus. In the simulation, the total length of synaptic delays along this loop was 15 ms, which, combined with membrane integration times, resulted in a total of approximately 25 ms/cycle, thus biasing the system toward 40-Hz oscillations.

Because intrinsic membrane oscillations have been reported, particularly in thalamic neurons [7], we also simulated a third type of model in which only a small subset of neurons were intrinsic cellular oscillators (the excitatory thalamic neurons, or 16.6% of the simulated cells), and all other neurons were of the “random spikes” type. The results, which appear on Figure 2E, indicate the presence of waxing-and-waning LFP oscillations within a much narrower band of the gamma range than in the model with nonoscillating integrate-and-fire neurons. Thus, a small proportion of intrinsic oscillators, in resonance with the delays associated with recurrent thalamocortical connectivity, suffices to generate spontaneous activity with precise characteristics.

### Facilitation of External Inputs by Spontaneous Activity

We then examined how spontaneous activity affects activation caused by external stimuli. To this end, we measured the number of spikes evoked during stimulation by a 500-ms depolarizing current pulse of variable intensity, while orthogonally varying the amount of ascending neuromodulatory current *I*_neuromodul_. This paradigm was applied both to a single neuron and to an entire thalamocortical column (in which case, only the thalamic excitatory neurons were stimulated), in both the intrinsic oscillator and the random spikes models (Figure 3).

**Figure 3:**
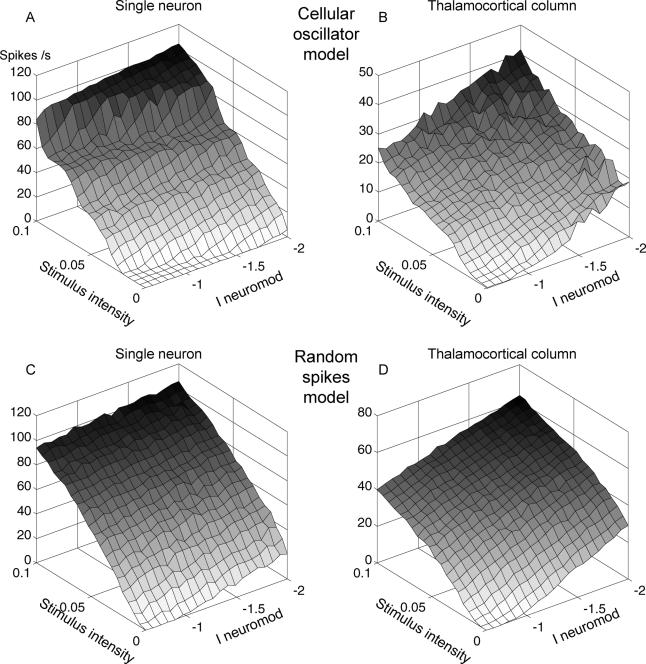
Combination of Intrinsic and External Activity in the Firing Rate of a Single Neuron or a Thalamocortical Column Simulations examined the joint effects of a variable external input (left axis [stimulus intensity], parameter *g*_input_, expressed in mS/cm^2^) and a variable neuromodulatory current that modified spontaneous activity and neuronal responsiveness (right axis [*I*_neuromodul_], expressed in μA/cm^2^). We simulated a single neuron (A and C) or a thalamocortical column (B and D), either with the cellular oscillator model (A and B) or with the random spikes model (C and D). In each case, we monitored the mean number of spikes emitted by pyramidal neurons during a 500-ms duration input.

In all cases, the results indicated a facilitating effect of ascending neuromodulatory afferences on external stimuli. The internal neuromodulatory current and the external input current combined in a smooth and largely additive fashion (Figure 3). Only for small values of the external current did the firing threshold cause a small nonlinearity: Small external inputs that failed to reach the threshold for firing when *I*_neuromodul_ was close to zero could be rescued and had an impact on firing rate when *I*_neuromodul_ was increased to 1.0–1.5 μA/cm^2^ (e.g., Figure 3D). Outside this special regime of very small external inputs, the results indicated that external inputs generally continue to be processed by a single neuron or thalamocortical column at all levels of the neuromodulatory current, albeit with a smooth, continuous increase in input efficacy as this neuromodulatory current is gradually increased.

We also performed simulations of the thalamocortical column while varying the duration of external stimulation. Those simulations showed that elevated firing rates are observed only for a temporal interval equal to the stimulus duration. As soon as the external stimulus is removed, a rapid decrease toward the basal spontaneous firing rate is seen. Thus, with the present values of the parameters, the recurrent thalamocortical connectivity alone is not sufficient to establish long-lasting sustained activity.

### Global Ignition of the Workspace by External Stimuli

We now describe how the response to external stimuli is radically changed once multiple columns are interconnected by long-distance connections into a global workspace. We simulated a multicolumn cortical model with four hierarchical levels, interconnected by long-distance corticocortical connections, and with two representations at each level. As in the previous section, spontaneous activity could be generated by intrinsic oscillations, random spikes, or a mixture of both; yet, those simulations again showed few differences, and we therefore only report the results of the intrinsic oscillation model.

In the previous section we saw how, in a single thalamocortical column, the neuronal activity caused by thalamic stimulation does not last much beyond the duration of stimulation. With the present parameters, the local circuitry of a column, although it includes excitatory loops, does not have sufficient strength to maintain activation over a durable interval. In the entire global workspace model, the effect of an identical stimulation is quite different. As show in Figure 4, stimuli of very brief duration elicit only a correspondingly brief pulse of activity, traveling in a feedforward manner through each of the thalamocortical columns. Crucially, however, there exists a critical stimulus duration beyond which activation begins to reverberate for a considerably longer duration (Figure 5A–5C). The existence of such a threshold is a characteristic of the dynamical system created by the recurrent bottom-up and top-down connectivity of the workspace. If the input is sufficiently long and strong, it is able to generate sufficient activation in the higher areas (i.e., areas labeled B, C, and D in Figure 1C), and those areas, in turn, send descending activation to lower areas, thus supporting the very activity that activated them in the first place. The result is the “ignition” of a coherent reverberating neuronal ensemble spanning across all areas (areas A–D in Figure 1C) and lasting 200–300 ms, as previously described [22]. The two successive processes of bottom-up propagation (proportional to stimulus duration) followed by top-down amplification and recurrent firing (incommensurate to stimulus duration) can be clearly seen as two successive firing peaks in the spike train of pyramidal neurons (Figure 5C, right-hand inserts).

**Figure 4:**
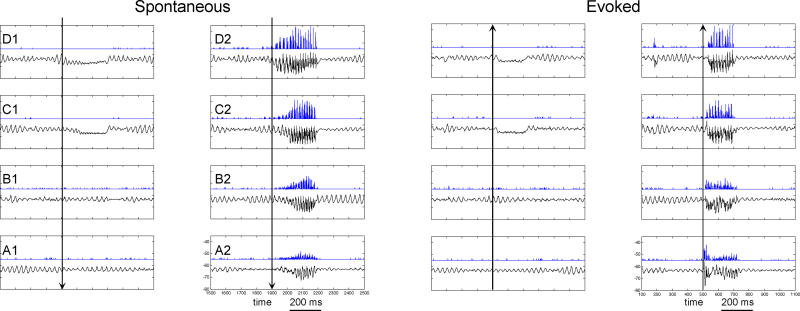
A Comparison of Spontaneous and Evoked Global Ignition in the Workspace Model Each tracing shows the temporal evolution of the LFP (bottom trace) and spikes (top trace) in one area of the model over a 1-s period, with *I*_neuromodul_ = −1.0 μA/cm^2^. In the two sets of tracings on the left, within an interval characterized by spontaneous membrane oscillations and low spike rates, the neurons coding for assembly 2 become spontaneously activated at an elevated rate for a period of 200–300 ms. The arrows mark the approximate onset of activation, which starts earlier from the higher areas D2 and C2, with a concomitant inhibition of spontaneous activity in areas D1 and C2. In the two sets of tracings on the right, the same network was stimulated by stimulus 2 at the time marked by the arrow. Note that activation now starts by a bottom-up propagation from areas A2 to D2, followed by top-down amplification in the reverse direction. This two-step process creates a characteristic firing profile with two successive peaks, particularly visible in area A2.

**Figure 5:**
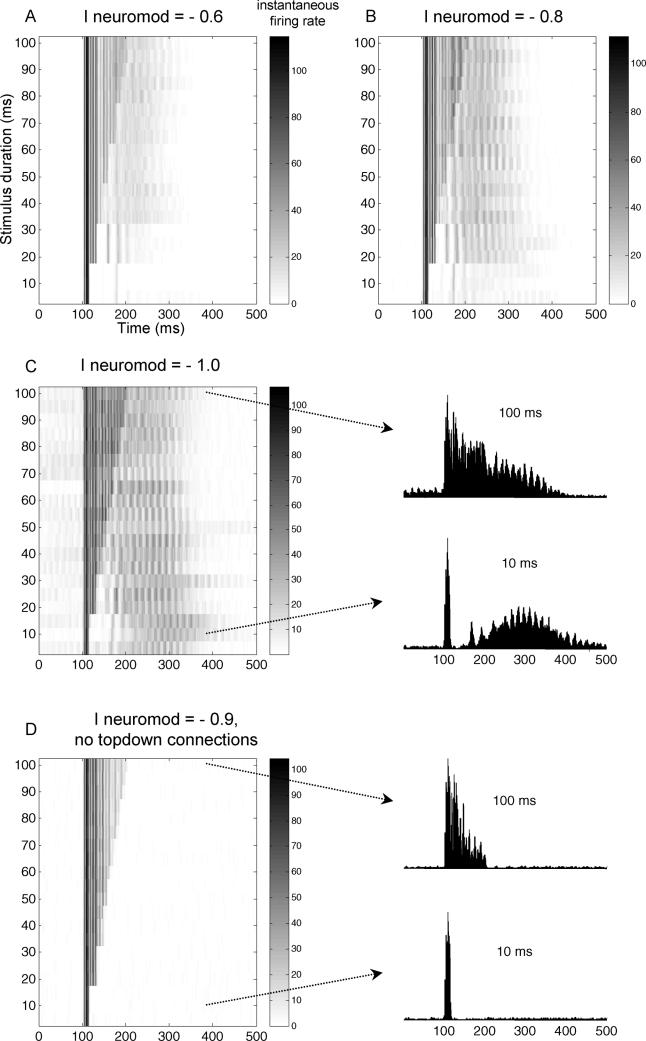
Reverberant Activity in the Global Workspace Model Imposes a Nonlinear Threshold on Incoming Stimuli Each panel shows the firing rate of pyramidal neurons in the lowest area A1 (coded by gray level) as a function of time (x-axis), in response to stimuli of variable duration (y-axis). Ignition is present when the duration of firing extends much beyond the duration of the stimulus, i.e., when a long tail of firing is present. In (A–C), the vigilance level—set by the ascending neuromodulation parameter *I*_neuromodul_—is progressively increased. This increase leads to a systematic change in the minimum duration necessary for ignition. Note that the figure is an average over 20 trials at each duration. Thus, the small trailing activation that can be seen in (A) and (B) even at durations below the threshold is due to a very small proportion of trials in which ignition did occur, due to stochastic variability, as further explained in Figure 6. The insets in (C) show the peristimulus-time histograms for stimulus of 10- or 100-ms duration, showing clearly the two firing peaks successively evoked by bottom-up activation and by top-down amplification. In a simulation in which top-down connections are disabled (D), the first peak is preserved, but the second peak is abolished.

During ignition of one representation, inhibition spreads to competing assemblies, yielding hyperpolarization and a suppression of ongoing oscillations (see Figure 4, third column). Thus, ignition occurs in a distributed but topologically delimited assembly—only a subset of neurons, forming a synchronous coding assembly, are active—but this assembly is surrounded by a broad context (or “penumbra” [38]) of temporarily inhibited neurons. In our previous simulations [22], we showed that this inhibition creates a competition that prevents more than one global representation from being active at the same time. Thus, if two stimuli occur in brief succession, the second one may fail to trigger global ignition, a feature akin to the classical psychological phenomenon of the “attentional blink” [39,40].

Ignition is a high-level collective phenomenon and is critically dependent on the integrity of long-distance recurrent connections. When top-down connections are disabled, as shown in Figure 5D, then ignition fails to occur and activation duration becomes exclusively proportional to stimulus duration, as in the single-column model. If top-down connection strengths are weakened, or if the connection probability is lowered, then a corresponding increase of the threshold duration for ignition is seen (up to a critical value beyond which ignition cannot occur, regardless of stimulus strength).

In a more complete model of cortical connectivity, global ignition would propagate to cortical areas beyond areas C and D, thus broadcasting the identity of the input stimulus to many cerebral processors. We therefore propose that the simulated process of ignition provides a minimal but plausible neural mechanism for the sudden access of sensory stimuli into conscious perception. The nonlinear dynamic threshold exhibited by the model may explain the sigmoid perceptual curve typically observed in psychophysical experiments using brief stimuli [41], and thus the very existence of a “limen” (threshold) of consciousness.

### Changes in Ascending Neuromodulation Affect the Ignition Threshold

In our model, the ignition threshold is not fixed, but can be changed by modifying the internal level of ascending neuromodulation. This is illustrated in Figure 5A–5C, showing the firing rate in response to stimuli of variable duration as the ascending neuromodulatory current *I*_neuromodul_ is changed (we obtained similar results when stimulus intensity, rather than duration, was manipulated). For *I*_neuromodul_ = −1.0 μA/cm^2^, even stimuli as short as 5 ms can be sufficient to trigger a long-lasting ignited state. For *I*_neuromodul_ = −0.8 μA/cm^2^, this access threshold is lowered to 15 ms, and for *I*_neuromodul_ = −0.6 μA/cm^2^, input stimuli of more than 30 ms are needed for global ignition. Interestingly, at this value of *I*_neuromodul_, there are no longer any ongoing spikes or gamma-band oscillations (see Figure 2). Thus, at this intermediate vigilance level, the model retains some capacity for a brief ignition by external stimuli, in the context of an otherwise quiet cortex. For even smaller levels of ascending neuromodulation, ignition is entirely abolished. Even very intense and durable external stimuli fail to elicit global ignition, and the evoked activation remains confined to the first thalamic and cortical steps in area A1. Those simulations demonstrate that a minimal level of spontaneous activity or “wakefulness” is necessary for the processing of external stimuli beyond the first few perceptual stages.

The effect of neuromodulation on the ignition threshold is mediated by at least two mechanisms. First, as described above (“Spontaneous Oscillatory Behavior in a Single Neuron and a Thalamocortical Column”), increasing neuromodulation brings neurons into spontaneous oscillatory states in which their membrane potential periodically approaches or reaches firing threshold, thus enhancing their capacity for prolonged firing. Second, as described in “Facilitation of External Inputs by Spontaneous Activity,” increasing neuromodulation also enhanced the neuronal response to an identical input. In the presence of recurrent connections, the two effects combine in a nonlinear manner, making it possible for the very same stimulus to lead either to a major ignition or to a small, bottom-up wave depending on the internal state of vigilance defined by the ascending neuromodulation parameter.

In summary, one role of ascending neuromodulation in the model is to modify, in a nonlinear manner, the capacity of external stimuli to enter into a long-lasting state of distributed representation: The workspace must be in the awake state before global access by any external stimulus can occur. Furthermore, the threshold values of the stimulus parameters beyond which ignition occurs appear very sensitive to changes in either ascending neuromodulatory influences or top-down connection strength. Our simulations therefore predict that measuring the perceptual threshold for brief stimuli should provide a good marker of the integrity and functional state of vigilance of the workspace system.

### Ignition as an All-or-None Stochastic Phenomenon

According to the model, the thalamocortical network is under a permanent state of spontaneous activity. Therefore, the processing of an identical external input may change with the local context of ongoing activation. For stimuli close to threshold, this “resonance” of external inputs with internal spontaneous activity plays a determinant role in allowing or blocking ignition. To demonstrate this point, we analyzed the impact of stimuli presented close to the ignition threshold by simulating 100 trials with an identical stimulus (15 ms duration) and a fixed, intermediate intensity of neuromodulation (*I*_neuromodul_ = −0.9 μA/cm^2^). We observed a considerable variability in neuronal responses, even in the first area, A1 (Figure 6). The first peak of bottom-up activation was present in a majority of trials, even in area D, although with some small variance in its exact onset and intensity (Figure 6A). However, the bulk of the variability occurred in the second peak, which could be completely absent or very intense and prolonged. In fact, the distribution of firing rates during this second peak was bimodal (Figure 6B). Thus, the variability actually betrayed an all-or-none stochastic process, with fluctuations in ongoing activity modifying, on a trial-by-trial basis, the probability of crossing the dynamic threshold for ignition.

**Figure 6:**
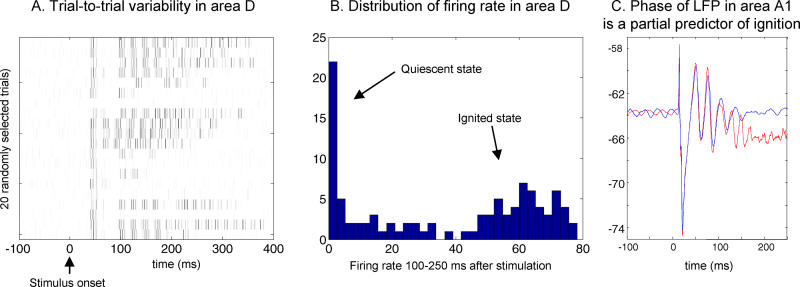
All-or-None Stochastic Fluctuations in the Processing of a Fixed Stimulus (A) Variability in sample spike trains evoked in area A1 by the same 15-ms stimulus, whose duration was close to ignition threshold (with *I*_neuromodul_ = −0.9 μA/cm^2^). Each of the twenty lines represents one trial. Note that the first peak is present on a majority of trials, while most of the variability affects the second peak. (B) Distribution of the mean firing rate in area A1 during the second peak (75–225 ms poststimulus), showing a bimodal distribution. (C) Mean local field potential in area A1, computed separately for trials that lead to ignition (firing peak > 40 spikes/s in the above time window) and for trials that did not (firing peak < 15 spikes/s). Note (1) the essentially identical stimulus-induced waves up to the sharp divergence about 110 ms poststimulus; and (2) the presence of a small but significant difference prior to the stimulus, which indicates that ignition is more likely to occur when stimulus presentation coincides with the depolarized phase of spontaneous ongoing oscillations.

Because our simulation is deterministic, ignition success or failure is entirely predictable from the neurons' current state and firing history. Yet, can it also be predicted from macroscopically observable variables? We classified trials as a function of whether ignition occurred or not, and then examined if those trials already differed in macroscopic variables such as the mean firing rate, mean local field potential, phase, or amplitude of spontaneous oscillations at the peak frequency within 100 ms prior to stimulus presentation. The only reliable predictor was the phase of spontaneous oscillations in area A1 (*p* < 0.045, Kolmogorov-Smirnov test). As illustrated in Figure 6C, ignition was more likely to occur when the first stimulus-evoked spikes reaching area A1 coincided with a period of relative depolarization, thus increasing the probability of spiking activity which could then propagate to higher regions.

Our simulations bear similarity to empirical observations by Fries et al. [42], who observed fluctuating, occasionally synchronized gamma-band oscillations in cat area V1 whose phase predicted the latency of firing in response to an external stimulus. Similarly, Super et al. [43] observed that the strength of prestimulus multiunit spiking activity in monkey area V1, and the correlation of this activity across multiple electrode sites, partly predicted whether the animal would report seeing a brief visual stimulus on a noisy background. Changes in baseline firing rate were not seen in the present analysis, but can be captured in our model by changes in the neuromodulatory input, which jointly affect baseline firing (see “Spontaneous Oscillatory Behavior in a Single Neuron and a Thalamocortical Column”) and ignition probability (see “Ignition as an All-or-None Stochastic Phenomenon”).

### Fixed Duration of the Ignited State

In our simulations, ignition typically lasts 200–300 ms. Why does it ever stop? Spontaneous cessation is due to the spike-rate adaptation current *I*_SRA_ in pyramidal neurons. This current leads to a progressive buildup of hyperpolarization, eventually overcoming the recurrent excitation provided by surrounded neurons. When the conductance *g*_SRA_ is forced to 0, thus preventing spike-rate adaptation, long-lasting activity lasts essentially ad infinitum. Choosing intermediate values of this parameter allows modulation of the duration of sustained activity. Thus, the typical duration of 200–300 ms observed in the present results depends on the particular choice of parameters and should only be taken as indicative. What is truly generic is that, for a given choice of parameters, interconnected columns excited by a brief suprathreshold stimulus enter into a temporary metastable state of global activity lasting for an approximately fixed duration, independent of the original stimulus duration.

Figure 7 illustrates what happens in simulations with stimuli whose duration extends beyond this fixed ignition duration. Two regimes of firing are seen: below 230 ms, activation lasts for a constant value (about 230 ms) independent of stimulus duration. Above 230 ms, activation duration becomes proportional to stimulus duration again, because the neurons are already adapted and are therefore unable to maintain any further sustained activity. These two regimes can be tentatively compared to the psychophysical observations of Efron [44], who asked subjects to estimate the onset and offset of brief visual stimuli of variable duration. He observed that below a critical value, subjects judged all stimuli to be of fixed duration (about 130 ms), independent of the actual duration of presentation. Beyond this value, judgments became more accurate and linearly related to stimulus duration (Figure 7B). The present simulations show that Efron's results are well captured, qualitatively at least, by a simple model, although the extent to which visual persistence is caused by local factors or by global loops as in the present simulation remains to be determined experimentally.

**Figure 7:**
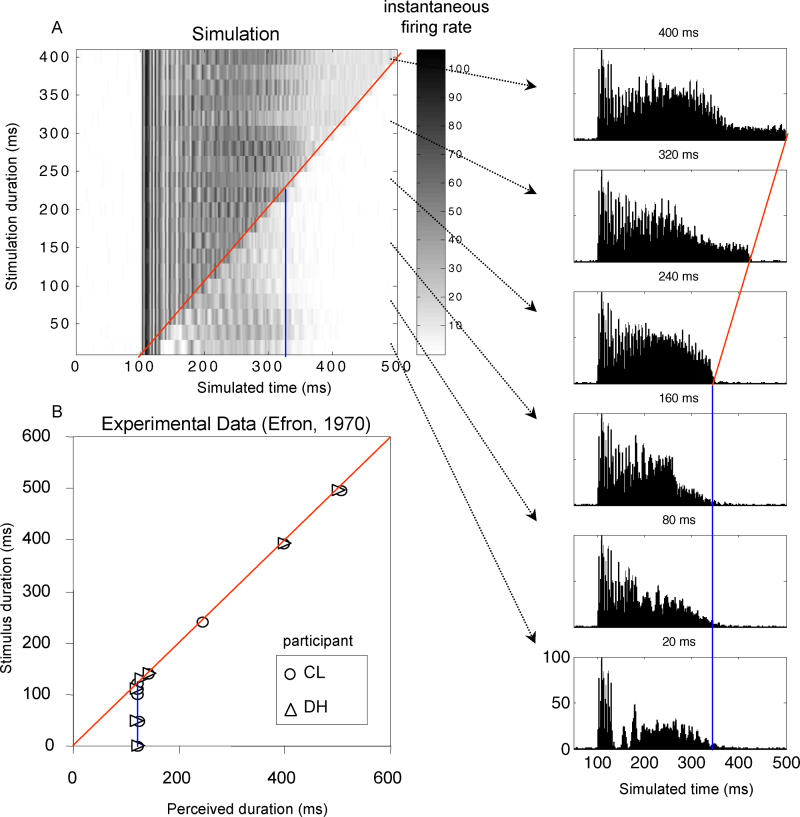
Duration of the Ignited State and Relation to “Visual Persistence” (A) Network response to stimuli of increasing duration (format as in Figure 5; *I*_neuromodul_ = −0.9 μA/cm^2^). Note how the duration of the ignition is initially constant and independent of the stimulus, then becomes proportional to it. (B) Empirical data on estimation of the subjective duration of a visual stimulus (replotted from data in Efron [44]). Both participants judged accurately the duration of stimuli above a critical duration, but judged duration to be constant below this value.

### Spontaneous Ignition

Up to now we have considered mostly the impact of an external stimulus onto the workspace system (ignition) and its interaction with ongoing oscillations of moderate intensity. However, simulations also revealed that ignition can occur spontaneously. In addition to waxing-and-waning gamma-band oscillations that are already present in the single-column model, the network with global long-distance connectivity occasionally falls into a state of globally synchronous elevated activity analogous to its ignition by external stimuli (see Figure 4). For a period of about 200–300 ms, the neurons coding for a given representation become spontaneously active synchronously within each of the four areas, with firing rates approaching 50–100 Hz. Meanwhile, the neurons coding for the other representation are quiescent and oscillations are actively suppressed in the higher areas C and D (see Figure 4, top left).

While this type of spontaneous states is highly similar to the ignited state evoked by external stimuli, a key difference is that during spontaneous ignition, elevated activity almost always starts at the highest level (area D), and then propagates downward to areas C, B, and A, where firing rates also tend to be lower. This is the reverse order of activation of that for external stimuli, and it underlines the top-down character of spontaneous ignition.

Another important difference is that spontaneous and evoked ignition tend to occur in different regimes of what we have referred to as vigilance. The above studies of ignition evoked by external stimuli were performed with −1.0 ≤ *I*_neuromodul_ ≤ −0.6 μA/cm^2^. In this regime, spontaneous ignition is quite rare, and spontaneous activity is mostly characterized by low firing rates and waxing and waning gamma-band oscillations, which do not interfere much with incoming external stimuli. Spontaneous ignition becomes a prominent phenomenon when *I*_neuromodul_ is of higher intensity (< −1.0 μA/cm^2^). For *I*_neuromodul_ < −1.2 μA/cm^2^, spontaneous activity becomes characterized by a steady stream of successive ignitions. When more than two global assemblies are present, the network successively visits them in random order. Thus, at high vigilance levels, the activity of workspace neurons resembles a “stream” (in the sense of William James) of discrete episodes of spontaneous, metastable, coherent activation separated by sharp transitions.

### Extinction of External Stimuli by Spontaneous Ignition

What happens to external stimuli when the workspace network is in a state of spontaneous global ignition? To study this, we first identified, in the absence of any external stimulation, an isolated period of spontaneous ignition of assembly 1 (Figure 8, center graphs; *I*_neuromodul_ = −1.0 μA/cm^2^). We then took advantage of the deterministic character of our simulation and re-ran the very same simulation, while presenting a second, competing stimulus in the input thalamic neurons of area A2, at various time lags relative to the spontaneous activation.

**Figure 8:**
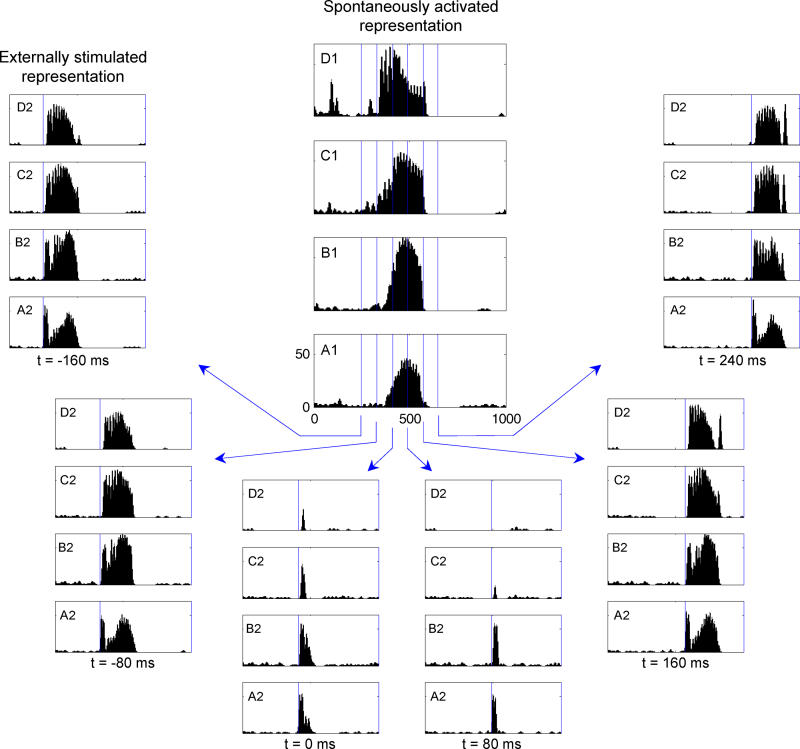
Competition between Spontaneous Workspace Activity and External Stimulation: A Possible Correlate of Inattentional Blindness In this simulation, a time period was selected during which the representation of stimulus T1 became spontaneously activated in the absence of any external stimulation (note how the activation starts at the higher areas C and D, and propagates top-down to areas B1, then A1). Starting from the same initial state, the simulation was then digitally reproduced with the addition of an external stimulation of stimulus T2 at various times relative to the peak of spontaneous activity in area D. When T2 occurred before or after the period of spontaneous T1 activity, a normal global reverberant activation of T1 ensued. However, when T2 occurred during spontaneous T1 activity, T2 processing was limited to a short, bottom-up wave through areas A2, B2, and, to a lesser and more variable extent, areas C and D. Such blocking of T2-induced activity, similar to the attentional blink [22], may represent the cerebral basis of a state of “inattentional blindness,” in which external stimuli fail to be detected during periods of spontaneous thought.

These simulations demonstrated a blocking effect of spontaneous activity. The stimulus presented during spontaneous ignition of a competing assembly does not lead to ignition, but is “extinguished” (Figure 8, bottom graphs). Activation caused by stimulus 2 initially propagates in a bottom-up manner through A2, B2, and occasionally even C2 and D2. Only the first peak is present, however: Competition prevents the establishment of a strong reverberating assembly, and thus of long-lasting metastable activity coding for stimulus 2. When this stimulus is presented either before or after the spontaneous ignition, however, no such blocking occurs, and full ignition is seen. Further, if the stimulus is presented just before the spontaneous ignition, the latter fails to occur. In summary, we observe an all-or-none competition between spontaneous and evoked activations, with either one capable of preventing the other. In the discussion, we suggest that this phenomenon may relate to inattentional blindness in humans.

## Discussion

### Predictions and Experimental Test of the Model

We have described the neuronal dynamics that emerge from a simplified model of multiple interconnected thalamocortical columns. This is an artificial and formal network, which by no means is anticipated to exactly reproduce the properties of actual thalamocortical networks, even in non-human species. Furthermore, although we have simulated a hierarchy of areas, each of them is very minimally implemented (one column for each representation), and the model therefore does not capture the increasingly abstract transformations that occur in actual cortical areas, nor the multiplicity of states they can have. Our purpose, however, is to examine to what extent the architectural principles and the cellular and molecular components introduced into the network may confer intrinsic dynamic properties that might be independent of the actual number of neurons and underlying complexity of the network. The correlations identified in the Results section between the present computer simulations and experimental data collected in humans and in non-human species legitimate, in our opinion, a close comparison with actual physiological and psychophysical experiments.

Two main states of the network have been observed: spontaneous gamma-band thalamocortical oscillations under the control of ascending neuromodulator systems; and, within a spontaneously active network, the sudden “ignition” of one out of many possible coherent states of high-level activity amidst cortical neurons with long-distance projections. In this discussion, we examine to what extent the delineation of these two dynamic states can capture empirical data on consciousness and its available neural correlates. We consider first how changes in spontaneous activity relate to the continuum of consciousness states that can be observed in the transitions between the awake state, sleep, anesthesia, or coma. Second, we discuss the all-or-none transition in neuronal activity associated with ignition and attempt to relate it to the transition from subliminal to conscious processing in various perceptual paradigms. Third, we discuss the interactions between vigilance and conscious access, and examine whether the model captures some of the processes underlying inattentional blindness.

### Spontaneous Thalamocortical Rhythms and States of Vigilance

The transition between the awake and asleep states is known to be regulated by various diffuse ascending neuromodulatory systems located in the brainstem, hypothalamus, and basal forebrain, and liberating substances such as acetylcholine, noradrenalin, serotonin, and histamine in the cortex and thalamus. In particular, cholinergic neurons in the pedunculopontine nucleus increase their firing prior to awakening and, through their diffuse projections, depolarize thalamic neurons, directly or indirectly, switching them out of the slow bursting mode and into fast gamma-band oscillations [9]. Similar effects can be obtained by electrical stimulation of the brain stem or by direct application of acetylcholine [45]. One mechanism for this involves the closing of a leaky K^+^ conductance coupled to muscarinic cholinergic and α_1_-adrenergic receptors. Moreover, constitutive gain-of-function mutations in the α_4_- and β_2_-subunit genes of the nicotinic acetylcholine receptor cause autosomal dominant frontal lobe epilepsy [46], and deletion of the β_2_-subunit is accompanied by a decrease of “microarousals” that take place during slow-wave sleep [47].

The present simulation incorporated only minimal details of these mechanisms. For simplicity, we merely modeled increases in vigilance by changes in a single current *I*_neuromodul_, summarizing the depolarizing influence of ascending neuromodulation systems onto thalamic and cortical neurons. Despite its extreme simplification, this mechanism was sufficient to generate a dynamic phase transition in our simulation, whose properties bear interesting similarities with actual empirical observations. We observed a threshold value of ascending neuromodulation beyond which structured neuronal activity emerged in the form of spontaneous thalamocortical oscillations in the gamma band (20–100 Hz, with a peak of the power spectrum around 40 Hz). This phenomenon was quite robust, since it was observed with two different mechanisms for spontaneous activity, either using intrinsic cellular oscillators or noisy spike generation (for other demonstrations of spontaneous oscillations in simpler network simulations, see [48,49]). This robustness appears to arise from the structure and delays inherent to thalamocortical loops, which function as a filter and selectively enhance gamma-band oscillations even when fed with unstructured noise.

The waxing-and-waning synchronous bursts of oscillations that we observed bear similarity with empirical observations. During the waking state, thalamic neurons exhibit fast spontaneous oscillations of their membrane potential at frequencies in the gamma band (20–80 Hz). These oscillations exhibit transient periods of thalamocortical resonance, which are detectable macroscopically as bouts of gamma-band oscillations using electrophysiological recordings, for instance in the cat thalamus and cortex [34,35], or in humans using electro- and magnetoencephalography [50]. When falling asleep, these fast and quickly changing rhythms disappear and are replaced by slower, more globally synchronized states of activity [9], including sleep spindles (7–14 Hz), followed in deeper stages of sleep by delta waves (1–4 Hz) and then slow-wave sleep (< 1 Hz). Our model did not incorporate mechanisms for generating such slow sleep rhythms, which could be usefully added in a more complete future simulation (see, for instance, [36]).

Following Llinas et al. [7], we propose that the spontaneous oscillatory activity that arises in thalamocortical networks constitutes the neuronal basis of the state of consciousness referred to as vigilance. An original feature of our simulation is to characterize precisely, in terms of a dynamic phase transition, the change in thalamocortical activity that characterizes a change in vigilance. Extending previous work by Wang [29], we show that there exists a vigilance threshold around which thalamocortical activity changes suddenly according to a Hopf bifurcation. The combination of continuity and discontinuity that this bifurcation presents may shed interesting light on the nature of the awakening process at the neuronal level. The Hopf bifurcation is continuous in the amplitude of spontaneous activity, which increases steadily from zero as vigilance increases. In that respect, our model incorporates a true continuum of consciousness states, from high vigilance to drowsiness and the various states of sleep, anesthesia, or coma. It is consistent with empirical observations that the ratio of high-to-low frequencies in the scalp EEG changes with the depth of anesthesia and correlates with objective tests of vigilance and reportability and with subjective measures of consciousness [51,52,53].

However, the Hopf bifurcation is also discontinuous in frequency space. As ascending neuromodulation increases, high-frequency activity appears suddenly, at a precise threshold, and within a well-defined frequency band (see Figure 2). This discontinuous jump in frequency space may capture the observation that during awakening or returning from anesthesia, there is a definite threshold for regaining of consciousness. According to our model, this threshold should coincide with the threshold for emergence of high-frequency, spontaneous thalamocortical oscillations. Note that, at the vigilance threshold, the thalamocortical high-frequency power drops quickly to zero. Thus, the precise value of the threshold might be difficult to locate empirically in noisy data. However, the Hopf bifurcation also predicts a continuous square-root increase in oscillation amplitude immediately above threshold (linear increase in power), a quantitative prediction that remains to be tested.

All the above properties of the Hopf bifurcation are generic, in the sense that they describe the behavior of a broad variety of dynamical systems [30]. Thus, they are likely to be independent of the particular details of the present simulation. Steyn-Ross et al. [54,55,56,57], on the basis of a formal mathematical exploration of a simplified cortical column, have also suggested that a phase transition occurs in cortical networks during anesthesia. While their model also predicts a collapse of the high-frequency content of the EEG, it differs from ours in predicting a discrete jump in EEG power. This is a testable difference between the two models.

We close this section by stressing that our model considers a highly simplified cortex in which oscillations are often apparent in the firing trains of single pyramidal cells. This may be considered a limitation of our approach, given that oscillations are rarely obvious in actual recording of awake animals (e.g., [58]). However, the basic properties of our model persist even in the absence of intrinsic cellular oscillators. In that case, oscillations are much less obvious in single-neuron firing trains, but are a network property most apparent in the local field potential. Furthermore, our simulations use only a small number of cells. As the number of neurons per cortical area is increased, oscillations become distributed and less conspicuous at the single-cell level. Overall, our simulations, although obviously very simplified, are not incompatible with experimental observations in awake cats, monkeys, and humans, which indicates that oscillations do occur in awake animals and humans, particularly under conditions of focused, effortful processing, and are typically more prominent in the local field potential than in the spiking activity of isolated cells [59,60,61,62,63,64,65,66,67].

It is also important to note that oscillations do not play a computational role in our model, which does not involve temporal coding. Oscillations and long-distance synchrony merely emerge as a consequence of the high level of reverberating activity in the network. Because oscillations are also present during local processing within a single thalamocortical column, high-frequency coherence and synchrony are expected to be partially ambiguous indicators of conscious access, present during both subliminal processing and conscious access, but with a higher intensity and over more distant sites during the latter.

### Anatomical Basis of the Awake State

Anatomically, our view predicts that, in the awake state, spontaneous activity is present in all areas, but exhibits a higher degree of organization (stream of discrete states) in higher cortical association areas, whose neurons are tightly interconnected at long-distance into a global neuronal workspace and mobilize other low-level areas in a top-down manner. Thus, we would expect cortical areas particularly rich in “workspace neurons” with long-distance connections (i.e., prefrontal, parietal, superior temporal, and cingulate cortices) to show the most intense and consistent spontaneous activity in the awake state. Other areas would also be active, but with more variability, reflecting the changes in contents contemplated by the subject at any given moment.

This prediction fits within a recent line of research that has examined the “resting state,” “default mode,” or “baseline” activity of the awake human brain at rest. This research has evidenced a broadly distributed network of areas active during rest, including dorsal and ventral medial prefrontal, lateral parietotemporal, and posterior cingulate cortices [12,14,68]. This network is not static and strictly confined, but constantly fluctuates in synchrony with changes in EEG spectral content [16]. Furthermore, prefrontal, parietal, and cingulate areas show the greatest drop in metabolism during various types of transitions away from the awake state, whether during anesthesia, sleep, coma, or the vegetative state [15,69,70,71,72,73]. Paus [74], reviewing several neuroimaging studies of vigilance in humans, suggests that a distributed neural circuit with nodes in the pontomesencephalic tegmentum, basal forebrain, thalamus, and anterior cingulate shows an identical decrease in activation whenever subjects fall asleep, are anesthetized, or exhibit a spontaneous drop in vigilance. This network fits well with the circuit predicted by our model, namely ascending neuromodulation systems, higher association cortices, and their associated thalamic nuclei.

Interestingly, Balkin et al. [73], studying the reestablishment of regional cerebral blood flow during awakening, have observed a two-stage process, whereby the brainstem and thalamus are restored first, later followed by an increase in prefrontal-cingulate activation and functional connectivity. This observations may correspond, in our model, to the two transitions observed as vigilance increases: first, waxing-and-waning LFP oscillations appear in thalamocortical columns, establishing a state of quiet vigilance with moderate spontaneous activity, but with a capacity for ignition by external stimuli; and second, at higher levels of the neuromodulation parameter, a stream of spontaneously ignited states appears, originating from higher cortical areas.

### Global Amplification and Conscious Access

Once the network was placed in the appropriate state of vigilance, our simulations examined its reaction to external stimuli. We observed a two-step process. All stimuli, even very brief ones, yielded a brief pulse of bottom-up excitation in the two areas of the model closest to the input side (areas A and B). However, for stimuli whose duration exceeded a threshold, we observed a propagation of firing into higher cortical areas C and D, and the establishment of a long-lasting reverberating state simultaneously involving bottom-up propagation and top-down amplification. We called this sudden, nonlinear self-amplification process “ignition” (as originally applied to Hebbian cell assemblies, see [75,76]). This term does not imply that the entire network becomes uncontrollably active. Rather, ignition reflects the activation of a topologically restricted assembly of neurons, distributed in many cortical areas, but all coding for a coherent mental object, and with joint inhibition of other neurons coding for competing objects.

Although we acknowledge that nonlinearities can occur at any stage in perceptual processing, including the retina, we speculate that the characteristic psychophysical sigmoidal curves observed when subjects report the perception of short or dim stimuli is related to the nonlinearity created by reverberant workspace interconnections. Psychological and neuronal models of sensory decisions assume a progressive integration of sensory evidence until the latter reaches a fixed threshold [77,78]. Our model proposes that this threshold is not implemented locally within a single area, but is a distributed property of many distant association areas. One ensuing prediction, recently verified experimentally using a dual-task paradigm, is that two independent integrations should not be able to proceed simultaneously [79]. In essence, the neuronal global workspace acts as the “central bottleneck” postulated in psychological models of dual-task processing.

The threshold for workspace ignition again reflects a bifurcation in dynamical system theory, but note that this bifurcation differs markedly from the one underlying the vigilance threshold. Conscious access, according to our model, is characterized by an all-or-none, first-order transition in which the relevant order parameters of the network (firing rates, gamma-band oscillations, synchrony) all show a discontinuous jump to higher values (whereas changes in vigilance generate a continuum of states, as described above).

Experimentally, the model predicts that each content of consciousness should be characterized by (1) activation of specific thalamocortical columns in relevant cerebral processors holding the conscious content (e.g., columns of neurons sensitive to motion in area MT, if the percept is one of motion); (2) joint activation of a broadly distributed subpopulation of neurons in prefrontal, parietal, and cingulate cortices; and (3) long-lasting reverberating activity among those sites, creating an episode of phase synchrony in the gamma band. Importantly, the model also predicts that for external stimuli that remain below the ignition threshold, the same specialized processors should be initially activated, but without leading to the subsequent global ignition.

Only a few studies to date have compared brain responses to comparable stimuli in conscious and nonconscious conditions. Those studies, obtained in diverse paradigms including masking [80], binocular rivalry [81], change blindness [82], attentional blink [83,84], and perceptual hysteresis [85] all suggest that the onset of conscious perception is associated with a sudden coactivation of parietal and frontal areas, often including the anterior cingulate [86]. Concomitantly, an amplification of activation in relevant posterior areas [80] is also seen during focal attention [87]. In EEG, bursts of induced gamma-band activity are observed during conscious access to a visual percept [88,89].

At the single-cell level, recordings in V1 and inferotemporal cortex in animals trained to report their visual percepts indicate that an initial phasic peak of firing is unaffected by reportability, but that reportable perception is associated with the presence of a late sustained activation that is not seen for nonreportable masked stimuli [90,91,92,93]. Thus, the firing histogram shows two successive peaks, with the second being present only during conscious perception, a profile highly similar to that observed in our simulations.

According to our model, the second peak, but not the first, should be affected by disrupting feedback from prefrontal, parietal, and cingulate cortices. This prediction could be tested by recording from visual cortices while reversibly cooling or inactivating distant association cortices. Note, however, that our model postulates a multiplicity of top-down loops, and it might not be possible experimentally to disrupt them all at once. Thus, such inactivation studies might not show a complete abolition of the ignited state, but only an elevation of the ignition threshold.

In our model, ignition places the thalamocortical network in a metastable state that lasts for a relatively fixed duration (e.g., 200–300 ms). Once an ignited assembly loses its support, the network again becomes available for either spontaneous or externally induced ignition. Thus, our simulations predict that multiple episodes of metastability should follow each other in a stream of discrete states, nested within a much slower fluctuation of vigilance. Few experimental studies are relevant this prediction. “Microstate” analyses have suggested that the human EEG consists of a series of quasistable states of 100–200 ms duration [94]. Likewise, Freeman has observed, in both awake cats and humans, sharp transitions in the phase of local potentials that occur simultaneously at distant sites across the scalp and delimit metastable periods of 100–300 ms duration [95]. At the single-cell level, hidden Markov models have been applied to the firing trains of simultaneously recorded neurons in the frontal cortex of a monkey performing a delayed-response task [96]. Those analyses revealed that neurons undergo a sequence of discrete states separated by abrupt changes, perhaps reflecting an internal rehearsal process. At present, however, these experimental forays remain isolated and have not yet been related to conscious reports.

### Interactions between Ongoing Spontaneous Activity and External Stimuli

An original aspect of the present simulations concerns the interactions between ongoing spontaneous activity and external stimuli. These interactions are mostly facilitatory: Higher spontaneous activity brings neurons closer to firing threshold, thus facilitating the detection of weak stimuli. However, very high spontaneous activity (spontaneous ignition) has a blocking role, preventing access to other external stimuli. Those two aspects lead to two distinct sets of predictions, which are considered in turn.

Concerning the first point, our model predicts that the threshold for conscious access (ignition) is not fixed, but decreases as vigilance increases. At one extreme, very low levels of vigilance prevent the possibility of ignition, even by long and intense stimuli. Such stimuli only lead to a short pulse of activation through the thalamus and areas A or sometimes B. Thus, we expect early sensory processing, but a lack of higher cortical processing, for sensory stimuli presented during altered states of consciousness. This prediction is consistent with empirical observations of auditory processing during sleep [97] or the vegetative state [98], where stimuli activated the thalamus and auditory cortex, but failed to generate the distributed state of correlated prefrontal, parietal, and cingulate activity observed in awake normal subjects. Similar observations have been made with tactile or pain stimuli, suggesting that the lack of prefrontal-parietal-cingulate ignition is quite characteristic of those states [99,100]. As described above, these areas also show a global decrease in their baseline metabolism even in the absence of stimuli, suggesting a considerable decrease in spontaneous activity. It would be interesting to relate, subject by subject, the resting metabolism and the activation by external stimuli of varying duration, to verify the predicted inverse correlation between spontaneous activity and the conscious access threshold.

We note that our model predicts the existence of an intermediate state in which the network has essentially no spontaneous activity, but is still capable of global ignition by intense and durable stimuli. This may tentatively capture some aspects of the “minimally conscious state,” where comatose subjects show fluctuating evidence for perception of environmental stimuli, and in whom distributed cortical activity, including cingulate cortex, can still be elicited by auditory or pain stimuli [101].

The inverse relation between vigilance and the conscious access threshold should also be present in the normal awake state, and could be manipulated empirically, for instance by sleep deprivation. Pharmacological agents such as nicotine, which can mimic and potentiate ascending cholinergic systems, might also have an influence on the perceptual threshold in visual masking or other psychophysical tests, which should be measurable both psychophysically and with brain imaging measures of ignition (e.g., prefrontal-cingulate activity in fMRI, P300 in event-related potentials). Finally, some disease processes might alter both spontaneous workspace activity and evoked ignition. In mice, deletion of the β_2_ subunit of the nicotinic receptor, which confers high-affinity acetylcholine or nicotine binding, leaves automatic navigation behavior intact, but interferes selectively with spontaneous exploration [102]. In schizophrenic patients, subliminal processing is intact, but the threshold for conscious perception of masked visual stimuli is increased, possibly relating to an impairment of top-down prefrontal-cingulate connectivity [103]. In such patients, we predict that nicotine might partially bring the conscious access threshold back toward its normal value. More generally, this threshold is predicted to provide a sensitive indicator of the anatomical integrity of top-down connections, and of the functional integrity of the vigilance system.

The facilitatory effects of spontaneous activity on the responsiveness to external stimuli are brought about by a simple mechanism that was observed in simulations of a single neuron or thalamocortical column: Larger membrane oscillations or impinging synaptic noise bring the neurons closer to firing threshold, where they are more sensitive to even small changes in their inputs. This mechanism has been demonstrated physiologically in cortical slices [104,105]. Our simulations show, however, that this phenomenon is mitigated by another, more collective phenomenon, which emerges only in the simulation of the globally connected model. At high levels of spontaneous activity, spontaneous ignition of structured global neuronal assemblies frequently happens and, during its occurrence, ignition by external stimuli is prevented (Figure 8).

Physiologically, there is some evidence for both of these aspects. First, optical imaging of visual cortex in anesthetized animals has revealed structured states of spontaneous ongoing activity, which have the same global organization as activity patterns evoked by external stimulation [10,11]. Second, high levels of spontaneous activity have been found to inhibit the sensory responses evoked by external stimuli, for instance by whisker deflection in somatosensory cortex [106]. It has been noted that such interactions with ongoing activity can provide an explanation for the large variability in spike trains evoked by the very same sensory stimuli [106,107]. Indeed, the present model predicts that there should be more variability in evoked spike trains in sensory areas during the normal awake state than during sleep or anesthesia.

The complete blocking of some incoming stimuli that occurs in our model offers a plausible explanation for the psychological phenomenon of “inattentional blindness” [26]. In this phenomenon, human observers engaged into an intense mental activity (such as detecting or counting stimuli of a certain type) become totally oblivious to other, irrelevant stimuli, even when they occur within the fovea for a long duration [27,28]. Although inattentional blindness is typically studied in the laboratory by placing subjects in a predefined task, our simulations suggest that spontaneous trains of thought, unrelated to external stimuli and instructions, may also exert a temporary blocking. Our model predicts that this state should be characterized by (1) an intense prefrontal-parietal-cingulate activation by the distracting thought or object prior to the presentation of the target stimulus; and (2) a proportional reduction of the target-induced activation to a brief bottom-up activation in specialized processors (Figure 8).

Only a single fMRI study to date provides direct support for prediction 2 [108]. However, there is considerably more evidence from the closely related phenomenon of the “attentional blink,” in which the distracting object is presented a few hundreds of milliseconds prior to the target stimulus. In particular, Marois et al. [109] obtained data compatible with prediction 1: the intensity of parietofrontal activity by the distracting task predicts the extent to which the target is extinguished. Furthermore, Marois et al. [83] supported prediction 2: during the blink, an unseen picture of house still activates the specialized parahippocampal place area, but fails to activate correlated activity in parietal, prefrontal, and cingulate normally observed in nonblinked trials. Similarly, in magnetoencephalography, the long-range synchrony linking frontal, temporal, and parietal areas is reduced for blinked stimuli [84]. Sergent et al. [110] used psychophysical methods to verify another prediction of the present model, namely the all-or-none character of conscious perception during the blink. Future research should extend those paradigms using time-resolved neuroimaging methods, such as event-related potentials, to test the prediction that early bottom-up activation is preserved, but later activation and top-down recurrent reverberation are suppressed in an all-or-none manner during the blink (see, for example, [111,112]).

### Comparison with Other Theories of Consciousness

The psychological concept of a “global workspace” was initially proposed by Baars [25] as a cognitive model of consciousness. Baars suggested that the global workspace can be neurally related to the nonspecific nuclei of the thalamus, particular the reticular formation, though he mentioned in passing that “it is possible that corticocortical connections should also be included” (p. 123 in [25]). The present model provides a much more detailed neurobiological implementation, and proposes that long-distance cortical connections are essential, particularly those linking prefrontal cortex to associative areas of the parietal and cingulate cortices.

A mention of parietoprefrontal networks in relation to consciousness can be found in Shallice [113] and Frith et al. [114]. In their pioneering efforts to specify the neural correlates of consciousness, Crick and Koch [115] also mentioned the importance of connections to and from prefrontal cortex (though more recently, Crick and Koch [38] defended the opposite view, that prefrontal cortex contributes to an “unconscious homunculus”). Their work, however, led them to the position that primary visual area V1 could not participate in conscious contents. Contrariwise, we see no fundamental reason why top-down mobilization would exclude primary areas, including V1, for instance in a high-acuity perception or mental imagery task.

Indeed, Lamme and his colleagues [116] have produced a wealth of empirical data indicating that late amplification of the local field potential in V1 correlates tightly with visual consciousness and attention in the awake monkey. Lamme suggests that consciousness occurs when recurrent bottom-up and top-down interactions occur between V1 and distant areas. The present model can be seen as a partial implementation of Lamme's hypothesis. One point of divergence is that we emphasize the unity of consciousness, involving a single ignited state that connects many distant areas, while Lamme [117] proposes that connections local to visual areas may be sufficient for a form of phenomenological awareness without conscious reportability (see also [118]).

None of the proposals reviewed so far were specified in terms of formal neural architectures that could simulate the dynamics of conscious versus nonconscious tasks. Tononi and Edelman [119] emphasized the role of information integration and of reentrant connections in establishing a “dynamic core” linking distributed cortical and thalamic neurons, yet without proposing an explicit simulation of this process. While the present work rests in part on a formalism established by Lumer et al. [120], their simulations focused on early and presumably nonconscious visual processing, with reentrant connections mostly linking cortical neurons in a horizontal manner rather than the present long-distance, top-down scheme for access to consciousness.

### Spontaneous Activity and Autonomy of the Organism

We close by noting that most theories of conscious processing have failed to recognize the important role of spontaneous thalamocortical activity (for exceptions, see [1,7,120]). By contrast, the present computational model proposes an essential role for spontaneous activity within an anatomically distinct set of “workspace neurons” in giving the formal organism an autonomy relative to the external world. Autonomy has several facets. First, in the absence of external signals, an autonomous organism must be capable of generating spontaneous representations and intentions (self-activation). Our present and past work [18,19,20] proposes that spontaneous neuronal activity is an essential component of this “projective style” [121,122], which departs from the input-output view currently dominant in the neural network community. The present work shows how sources of noise such as spontaneous membrane oscillations and noisy synaptic transmission can be harnessed to generate a stream of highly organized states of self-activation in the complete absence of external inputs. This capacity is expected to play a crucial role in the spontaneous generation of novel, flexible behavior, as evidenced for instance in neuropsychological tests such as the Tower of London test or the Wisconsin card sorting test.

Second, even when submitted to external stimulation, an autonomous organism must be capable of representing internally only those stimuli that are relevant to the present situation. Relevant stimuli must first be selected, based on reward-based evaluation systems not included in the present model (but see [123]), and then maintained online over a time period often incommensurate with their actual input duration, all the while resisting distraction by irrelevant stimuli. The present work provides the basic building blocks for such a decoupling of part of the organism's internal activity from its current inputs. A crucial role is attributed to the strongly recurrent connectivity of cortical neurons in association areas, which collectively form a conscious workspace, an internal “milieu” buffered from the outside world, and within which mental hypotheses can be entertained and discarded at will. An inevitable consequence of this autonomy, however, is that some stimuli are inappropriately filtered out, thus causing inattentional blindness.

## Materials and Methods

### 

The general architecture of the neural network that forms the basis of the present simulations has been described elsewhere [22]. Here we expand upon this description by providing new specifications relevant to the generation of spontaneous activity.

#### Model neurons

Neurons are modeled as single-compartment integrate-and-fire units with membrane potential *V*. When *V* exceeds a threshold θ, a spike is recorded and transmitted to other neurons with a delay, and *V* is reset to −80 mV for a refractory period of 4 ms.

The temporal evolution of *V,* expressed in millivolts, is given by:


*C* d*V*/d*t* = − *g*_Leak_ (*V* − *V*_Rest_) − *I*_NaP_ − *I*_KS_ − *I*_GABA_ − *I*_AMPA_ − *I*_NMDA_ − *I*_SRA_ − *I*_input_ – *I*_neuromodul_(1)

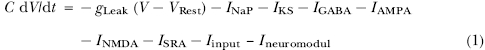



where *C* = 1 μF/cm^2^ and currents are expressed in μA/cm^2^. The leak conductance is *g*_Leak_ = 0.1 mS/cm^2^, so that the membrane time constant *C*/*g*_Leak_ is 10 ms.

*I*_GABA_, *I*_AMPA_, and *I*_NMDA_ are the total synaptic currents, respectively, from fast GABAergic, AMPA and NMDA glutamatergic synapses [120]:



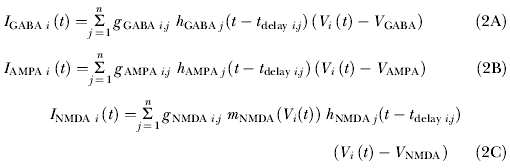



where the sum in each case is over all relevant neurons connected to postsynaptic neuron *i* (inhibitory interneurons for *I*_GABA_, bottom-up and intra-column excitatory neurons for *I*_AMPA_, and top-down excitatory neurons for *I*_NMDA_). The *g_i,j_* are the synaptic strengths and *t*_delay *i,j*_ the transmission delays in milliseconds from neuron *j* to neuron *i*. The functions *h_j_*(*t*) characterize the synaptic inputs from neuron *j* and are given by the convolution of the spike train *s_j_*(*t*) with the synaptic activation profile *a*(*t*):


*h_j_* (*t*) = *s_j_* (*t*) o× *a*(*t*)(3)

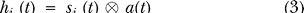



where *a*(*t*) = α (e^−*t*/τ1^ − e^−*t*/τ2^) / (e^−*t*peak/τ1^ − e^−*t*peak/τ2^), with *t*peak = τ_1_ τ_2_ / (τ_1_ − τ_2_), where τ_1_ and τ_2_ are rise and decay constants characteristic of each channel [120]. In the NMDA case only, the effective conductance is scaled by *m*_NMDA_(*V*), a factor that characterizes the voltage dependence of NMDA channels [37]:


*m*_NMDA_(*V*) = (1 + 0.280 e^−*V*/16.1^)^−1^ (4)

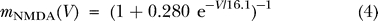



*I*_SRA_ is an additional current used to model spike-rate adaptation [37] that is present only in cortical pyramidal neurons:


*I*_SRA_ = *g*_SRA_ (*V* − *V*_SRA_)(5)

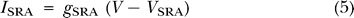



After each spike, the conductance *g*_SRA_ is increased by a small quantity (here 0.01 mS/cm^2^); otherwise, it decays exponentially according to τ_SRA_ d*g*_SRA_ /d*t* = −*g*_SRA_.

*I*_input_ is the current applied during stimulus presentation to thalamic neurons of the lowest hierarchical level to simulate a visual or auditory input:


*I*_input_ (*t*) = *g*_input_ (*V*(*t*) − *V*_AMPA_)(6)

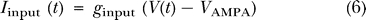



A final current, *I*_neuromodul_, summarizes the known depolarizing effects of ascending activating systems such as those from cholinergic, noradrenergic, and serotoninergic nuclei in the brain stem, basal forebrain, and hypothalamus [124]. This parameter is used to control the level of vigilance.

#### Origins of spontaneous activity

We consider two possible sources for spontaneous activity, both of which are meant as theoretical idealizations on a continuum of possibilities. The first case, hereafter referred to as the “cellular oscillator model,” corresponds to our initial publication [22] and is a purely deterministic model in which neurons follow simple differential equations incorporating persistent sodium and slowly inactivating potassium currents whose interplay generates intrinsic gamma-band oscillations of membrane potential [29], comparable to those recorded experimentally [7]. In this cellular oscillator model only, *I*_NaP_ and *I*_KS_ are persistent sodium and slowly inactivating potassium currents [29] whose simplified expression is


*I*_NaP_ = *g*_NaP_*m*_NaP_ (*V* − *V*_Na_)(7)

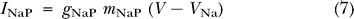



where *m*_NaP_ = (1 + e^−(*V*+51)/5^)^−1^, and


*I*_KS_ = *g*_KS_*m*_KS_ (*V* − *V*_K_) (8)

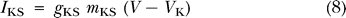



where τ_KS_ d*m*_KS_/d*t* = (1 + e^−(*V* + 34)/6.5^)^−1^ − *m*_KS_, with τ_KS_ = 6 ms. We also describe another simulation, hereafter called the “random spikes model,” in which intrinsic membrane oscillations are absent (*I*_NaP_ = *I*_KS_ = 0), but stochastic spontaneous activity arises from fast, random fluctuations in membrane potential, capturing the joint effects of synaptic and postsynaptic noise on spike initiation. Thus, in the random spikes model, on each time step the spike threshold θ varies randomly, independently for each neuron, as a Gaussian with mean −48 mV and standard deviation 5 mV, while in the deterministic cellular oscillator model, the spike threshold θ is fixed at −48 mV.

#### Columnar organization and connectivity

Each thalamocortical column comprised 80 excitatory and 40 inhibitory neurons organized in a three-layered structure, schematizing supragranular, infragranular, and layer IV cortical neurons, and a corresponding thalamic sector (see Figure 1C). Within each layer, there were 20 excitatory and 10 inhibitory neurons.

Connections were established with a 60% probability. Connection parameters (synaptic strength γ, expressed in mS/cm^2^; and transmission latency *t*_delay *i,j*_ *,* expressed in ms) were drawn from Gaussian distribution with 10% standard deviation around a fixed mean. All neurons connecting to a given area randomly contacted excitatory and inhibitory neurons with the same probability and strength.

Concerning inhibition, a broad variety of inhibitory cell types are known to exist in cortex. Here, we adopted a simplified scheme in which inhibitory neurons sent only horizontal connections to other neurons within their cortical layer of origin, both within a column (γ_GABA_ = 0.12, τ_delay_ = 2) and toward other competing columns within the same area (γ_GABA_ = 0.60, τ_delay_ = 2).

Concerning the intracolumnar connectivity, we adopted principles and parameter values that captured the major properties of translaminar connections [120,125], although we did not attempt to capture the possible functional roles of the different layers (e.g., see [126]). Latencies were taken from Lumer et al. [120], which describes how they must be adjusted to take into account the coarseness of the present three-layer model and the fact that, in reality, some of these pathways are disynaptic. Thalamic excitatory neurons projected to layer IV (γ_AMPA_ = 0.20, τ_delay_ = 3) and, with lesser strength, to infragranular neurons (γ_AMPA_ = 0.10, τ_delay_ = 3). Layer IV excitatory neurons projected to supragranular neurons (γ_AMPA_ = 0.15, τ_delay_ = 2). Supragranular excitatory neurons projected to infragranular neurons (γ_AMPA_ = 0.10, τ_delay_ = 2). Finally, infragranular excitatory neurons projected to layer 4 (γ_AMPA_ = 0.05, τ_delay_ = 7), to supragranular neurons (γ_AMPA_ = 0.05, τ_delay_ = 7), and to the thalamus (γ_AMPA_ = 0.075, τ_delay_ = 8).

Concerning corticocortical projections, supragranular excitatory neurons of each area projected to layer IV of the next area (γ_AMPA_ = 0.05, τ_delay_ = 3). In agreement with physiological observations [127,128], top-down connections were slower, more numerous, and more diffuse. They connected the supra- and infragranular excitatory neurons of a given column to the supra- and infragranular layers of all areas of a lower hierarchical level (see Figure 1B). Strong top-down connections linked columns coding for the same stimulus (γ_NMDA_ = 0.05), while weaker top-down connections projected to all columns of a lower area (γ_NMDA_ = 0.025). Transmission delays increased with cortical distance (τ_delay_ = 5 + 3δ, with δ = 1 for consecutive areas, δ = 2 for areas two levels apart in the hierarchy, etc).

#### Parameter values

All parameter values were fixed and adopted without modification from [29,37,120]: *V*_Rest_ = −67 m*V*, *V*_Na_ = 55 mV, *V*_K_ = −90 mV, *V*_GABA_ = *V*_SRA_ = −70 mV, *V*_AMPA_ = *V*_NMDA_ = 0 mV; α_GABA_ = 0.175, α_AMPA_ = 0.05, α_NMDA_ = 0.0075; τ1_GABA_ = 1 ms, τ1_AMPA_ = 0.5 ms, τ1_NMDA_ = 4 ms, τ2_GABA_ = 7 ms, τ2_AMPA_ = 2.4 ms, τ2_NMDA_ = 40 ms, τ_SRA_ = 200 ms; and *g*_input_ = 0.06 mS/cm^2^. To avoid an artificial digital reproducibility of oscillations across neurons, the following parameters were generated randomly for each neuron, from a Gaussian distribution with a 5% standard deviation and the following mean: *g*_NaP_ = 0.2 mS/cm^2^, *g*_KS_ = 8 mS/cm^2^. To study variations in the state of spontaneous activity, *I*_neuromodul_ was treated as the only experimental variable between 0 and −2 μA/cm^2^.

Two main measures were used to describe network activity: the mean number of spikes emitted per millisecond, and the mean membrane potential, both averaged across all pyramidal cells in a given column. For simplicity, we refer to the latter parameter as the LFP. Note, however, that actual electrophysiological recordings of the LFP are much more complex, in at least two respects: (1) LFPs mainly reflect postsynaptic potentials rather than spike activity; and (2) they vary across cortical layers. Given this complexity, we opted for the simplest possible summary measure of the collective evolution of the network; however, we verified that our results still held when other measures were used, for instance the mean synaptic inputs of pyramidal cells within a specified cortical layer.

## Abbreviations

EEG: electroencephalogram
LFP: local field potential
